# BDMC protects AD *in vitro* via AMPK and SIRT1

**DOI:** 10.1515/tnsci-2020-0140

**Published:** 2020-09-09

**Authors:** Chenlin Xu, Zijian Xiao, Heng Wu, Guijuan Zhou, Duanqun He, Yunqian Chang, Yihui Li, Gang Wang, Ming Xie

**Affiliations:** The First Affiliated Hospital, University of South China, Hengyang, Hunan 421001, People's Republic of China; Xiangxi Autonomous Prefecture People’s Hospital, Jishou, Hunan 416000, People's Republic of China; Department of Rehabilitation, Union Hospital, Tongji Medical College, Huazhong University of Science and Technology, Wuhan, Hubei 430022, People's Republic of China

**Keywords:** Alzheimer’s disease (AD), bisdemethoxycurcumin (BDMC), AMPK, SIRT1

## Abstract

**Background:**

Alzheimer’s disease (AD) is a common neurodegenerative disorder without any satisfactory therapeutic approaches. AD is mainly characterized by the deposition of β-amyloid protein (Aβ) and extensive neuronal cell death. Curcumin, with anti-oxidative stress (OS) and cell apoptosis properties, plays essential roles in AD. However, whether bisdemethoxycurcumin (BDMC), a derivative of curcumin, can exert a neuroprotective effect in AD remains to be elucidated.

**Methods:**

In this study, SK-N-SH cells were used to establish an *in vitro* model to investigate the effects of BDMC on the Aβ_1–42_-induced neurotoxicity. SK-N-SH cells were pretreated with BDMC and with or without compound C and EX527 for 30 min after co-incubation with rotenone for 24 h. Subsequently, western blotting, cell viability assay and SOD and GSH activity measurement were performed.

**Results:**

BDMC increased the cell survival, anti-OS ability, AMPK phosphorylation levels and SIRT1 in SK-N-SH cells treated with Aβ_1–42_. However, after treatment with compound C, an AMPK inhibitor, and EX527, an SIRT1inhibitor, the neuroprotective roles of BDMC on SK-N-SH cells treated with Aβ_1–42_ were inhibited.

**Conclusion:**

These results suggest that BDMC exerts a neuroprotective role on SK-N-SH cells *in vitro* via AMPK/SIRT1 signaling, laying the foundation for the application of BDMC in the treatment of neurodegenerative diseases related to AMPK/SIRT1 signaling.

## 1 Introduction

Alzheimer’s disease (AD), a severe and progressive brain disorder, is predicted to increasingly affect a significant portion of the aging human population worldwide [1]. AD is characterized by the accumulation of β-amyloid protein (Aβ) plaques, intracellular tangles and the loss of neurons in selective brain regions [2]. To date, available therapies such as cholinesterase inhibitors (including donepezil, galantamine and rivastigmine) and *N*-methyl-d-receptor antagonists (including memantine) [3] only alleviate disease symptoms of AD, and no effective therapeutic approaches were found to address the underlying pathological processes [4]. Consequently, discovering more effective drugs and uncovering the pathological mechanisms are urgent medical needs related to the treatment of AD.

Attempts to target individual molecules that may reduce the pathological impact of AD have been made to counteract cell death by the introduction of survival-proactive molecules. One of these molecules that we have studied is bisdemethoxycurcumin (BDMC), a natural derivative of curcumin, due to its ability to protect against Aβ neurotoxicity in AD [5,6]. However, its therapeutic potential is limited due to poor bioavailability, low solubility in aqueous media and poor pharmacokinetic profiles [7,8,9,10,11]. BDMC, the most potent and stable curcuminoid in biological systems [12,13,14], is produced based on the curcumin matrix by removing the 3-position methoxy group on the bilateral benzene ring and retaining the 4-positionhydroxyl group [15]. It was reported that BDMC exerts more promising pharmacological and biological effects than curcumin [16]. BDMC also shows a more potent apoptotic effect via downregulating the levels of heme oxygenase-1 and BCL-2 and upregulating the production of reactive oxygen species (ROS) compared with curcumin [17]. BDMC, with its higher polarity, hydrophilicity, water solubility, increased stability and improved nuclear cellular uptake than the parent curcumin, owns considerable anti-oxidant, anti-inflammatory and antiproliferative effects [14,18], which may be a more promising drug for clinical application.

In AD, Aβ is neurotoxic and initiates the hyperphosphorylation of tau, resulting in the dysfunction and the death of neurons [19]. In addition, there is an increase in oxidative stress (OS) in response to the increased Aβ levels [20], and this is considered to be an early event in the AD development [21,22]. Numerous types of signaling pathways may be associated with the pathological process of AD. Attempts to target signaling pathways that are associated with the pathological process of AD have been made to counteract OS and Aβ-induced cell death. One of the signaling pathways is AMP-activated protein kinase (AMPK), a key kinase associated with regulating cell energy metabolism, modulating the generation of Aβ [23] and exerting an essential role in energy homeostasis via responding to low cellular energy [24]. AMPK has also been revealed to have beneficial functions in cells including anti-oxidant, antitumor and anti-inflammation activities [25,26]. Another signaling pathway involved is silent information regulator 1 (SIRT1), an NAD^+^-dependent histone deacetylase, which has been shown to decrease aging and the progression of age-related disorders. An increase in OS during aging could also decrease the catabolic activity of SIRT1, possibly by ROS production [27]. Moreover, activated SIRT1 can control OS and generate neuroprotective effects [28]. Therefore, the AMPK-SIRT1 pathway may be potentially involved in the pathogenesis of AD.

The aim of this study is to investigate the effects of BDMC on SK-N-SH cells treated with Aβ_1–42_, the major component of amyloid plaques that is prominently increased in the human AD brain [29], and the underlying mechanisms. The results indicate that BDMC may exert a cell-protective role in SK-N-SH cells against Aβ_1–42_-induced neurotoxicity via AMPK/SIRT1 signaling.

## 2 Materials and methods

### 2.1 Synthesis of BDMC

Bisdemethoxycurcumin (BDMC, containing >80% curcumin and >94% curcuminoid content purity) purchased from Sigma-Aldrich (St. Louis, MO, USA) was dissolved in dimethyl sulfoxide (DMSO) and added to the cell culture medium for drug treatment in *in vitro* experiments according to our previous study [30].

### 2.2 Oligomeric Aβ_1–42_

Oligomeric Aβ_1–42_ was prepared in our lab according to the previous study [31]. Briefly, 1 mg of synthetic Aβ_1–42_ powder (1932-2-15, Shanghai Qiangyao Biological Technology, Shanghai, China) was dissolved in 22 µL DMSO and then diluted into Dulbecco’s modified Eagle’s medium (DMEM/F-12, Thermo Scientific Hyclone, Beijing, China) to produce a nontoxic concentration of 0.1% DMSO. The diluted solutions were incubated for 24 h at 4°C and centrifuged at 14,000 × *g* for 10 min. The supernatant was collected and used as 1 mM oligomeric Aβ_1–42_ for cell culture experiments.

### 2.3 Cell culture and treatments

SK-N-SH cells were cultured as previously described [32]. SK-N-SH cells purchased from Shanghai FUHENG Biotechnology Co., Ltd (FH0164, Shanghai, China) were cultured in DMEM/F-12 supplemented with 10% fetal bovine serum (Sijiqing Biotech Corp.) and 100 U/mpenicillin/streptomycin (ps) mixture (Solarbio Biotech Corp.) in 75 cm^2^ cell culture plates (Corning Inc.) at 37°C in a humidified 5% CO_2_ atmosphere.

To investigate the effects of BDMC on SK-N-SH cells treated with Aβ_1–42_, 1 × 10^4^ SK-N-SH cells were seeded into 96-well cell culture plates (for cell viability assays) or 24-well cell culture plates (for SOD, GSH and western blot assays) and treated as follows: (1) cells were treated with 15 µM BDMC for 30 min following 24 h co-culture with Aβ_1–42_; (2) cells were treated with 10 µM compound C and 15 µM BDMC for 30 min following a 24 h co-culture with Aβ_1–42_; (3) cells were treated with 100 nM EX527 and 15 µM BDMC for 30 min following a 24 h co-culture with Aβ_1–42_. Subsequently, cell viability, SOD, GSH and western blot assays were performed.

### 2.4 Western blotting

Western blotting was performed as described in the previous studies [33,34,35] with minor modifications. Protein samples were separated via 10% SDS-PAGE and electroblotted onto polyvinylidene difluoride membranes (Millipore) for 3 h at 300 mA. The membranes were blocked with 5% nonfat dry milk or BSA dissolved in Tris–HCl saline buffer containing 0.1% Tween-20 (TBS-T, PH 7.4). Subsequently, the blots were incubated overnight at 4°C with one of the following antibodies: rabbit anti-p-AMPK (1:500; cat. no. ab23875; Abcam, USA) and rabbit anti-sirt1 (1:500; cat. no. ab220807; Abcam, USA). Membranes were washed three times for 5 min each time in TBS-T. HRP-coupled goat antirabbit secondary antibodies (1:1,000; Boster, Wuhan, China) diluted in TBS-T were then applied for 1 h. Membranes were washed three times in TBS-T for 5 min each time at room temperature. Immunoreactive signals were then visualized with the enhanced chemiluminescence solution (Bio-Rad, USA). Signal intensities were quantified by densitometric analysis using ImageJ software (Dental Diagnosis Science, San Antonio, TX).

### 2.5 MTT assay

MTT assay was performed as previously described [36,37,38] with minor modifications. At the indicated time points, SK-N-SH cells were maintained in culture medium supplemented with 10 µL 3-(4,5-dimethyl-2-thiazolyl)-2,5-diphenyl-2-*H*-tetrazolium bromide (MTT, 500 µg/mL) (cat. no. M1020; Solarbio, China) for 4 h. Following aspiration of the culture medium, 100 µL DMSO was added to each well in the culture plates, and the cells were incubated at 37°C for 30 min. Optical density was measured spectrophotometrically at a wavelength of 410 nm.

### 2.6 Measurement of SOD activity

The WST-1 Cell Proliferation Assay kit was used to detect the SOD activity according to the manufacturer’s instructions (cat. no. A001-3-2; Jiancheng Biotech Ltd, Nanjing, China) [39,40]. The xanthine–xanthine oxidase system was applied to produce superoxide ions, which can react with 2-(4-iodophenyl)-3-(4-nitrophenol-5-phenlyltetrazolium chloride) to form a red formazan dye. The absorbance was determined at a wavelength of 550 nm. Protein concentration was determined using a BCA protein assay kit (QPBCA, Sigma-Aldrich, USA). The values were expressed as units per mg protein. One unit of SOD was defined as the amount of SOD inhibiting the rate of reaction by 50% at 25°C.

### 2.7 Measurement of GSH

GSH measurement was performed according to the previous method with minor modifications [41]. SK-N-SH cells in culture medium were centrifuged at 500 × *g* for 10 min and washed twice with PBS. The collected SK-N-SH cells were resuspended in the protein removal reagent and vigorously vortexed. Subsequently, the samples were rapidly frozen and thawed with liquid nitrogen twice at 37°C followed by incubation at 4°C for 5 min. After centrifugation at 10,000 × *g* for 10 min, the supernatant was collected. The GSH levels were determined using a GSH and GSSG assay kit (cat. no. S0053; Beyotime Institute of Biotechnology, Shanghai, China) according to the manufacturer’s instructions.

### 2.8 Statistics

Data were represented as the mean ± SEM. Comparison between groups was performed using Student’s *t*-test for independent samples using SPSS 18.0. Statistical significance was considered when *P* < 0.05.

## 3 Results

### 3.1 BDMC enhances AMPK phosphorylation and SIRT1 expression levels in SK-N-SH cells treated with Aβ_1–42_

To investigate the effects of BDMC on AMPK phosphorylation and SIRT expression in SK-N-SH cells induced by Aβ_1–42_, western blotting was performed after the cells were pretreated with BDMC and co-cultured with Aβ_1–42_ for 24 h.

**Figure 1:**
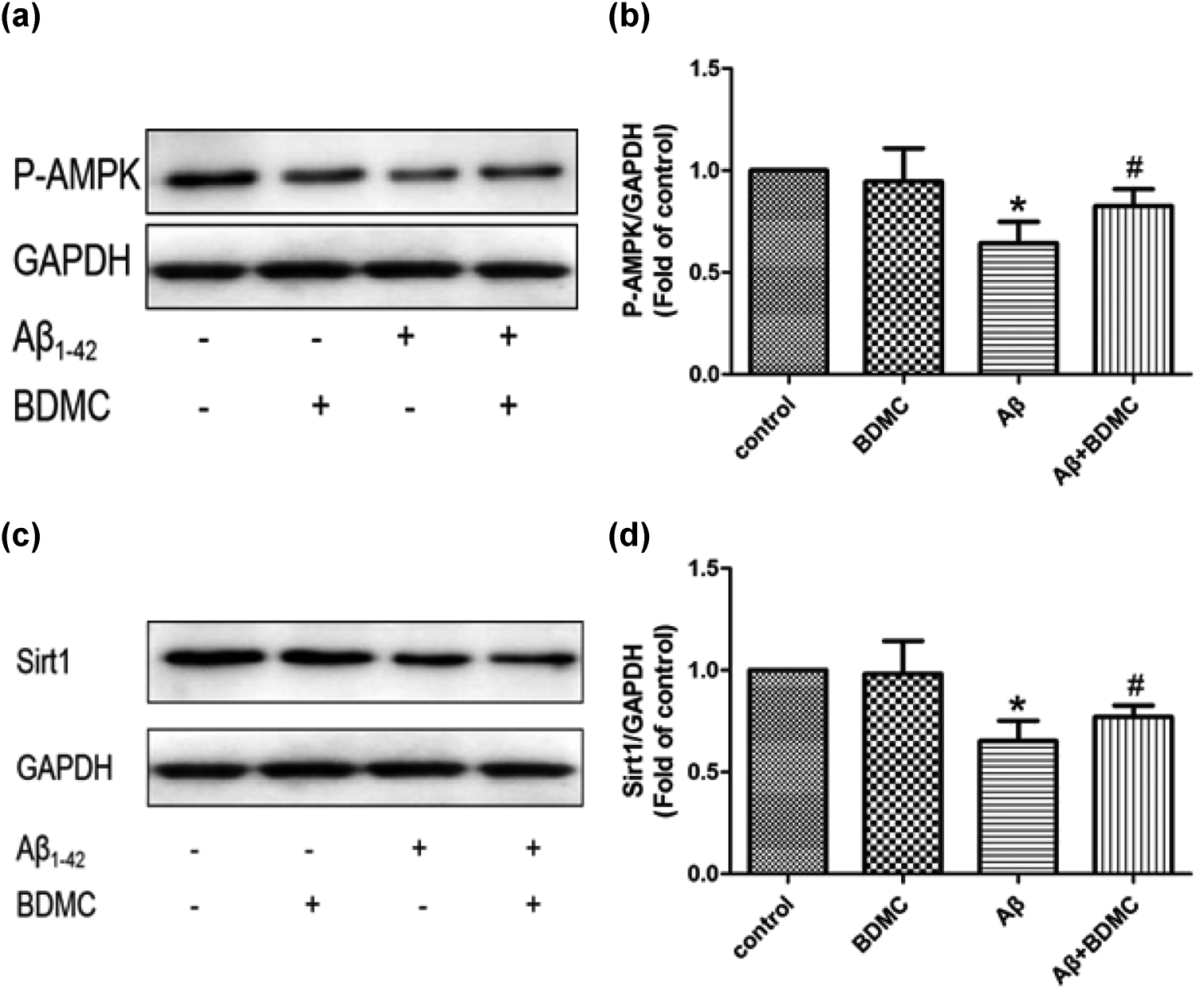
Effects of BDMC on AMPK phosphorylation and SIRT1 expression levels in SK-N-SH cells induced by Aβ_1–42_. A total of 1 × 10^4^ SK-N-SH cells were pretreated with BDMC at a concentration of 15 µM for 30 min before 24 h co-culture with Aβ_1–42_. Western blotting was then performed to assess the AMPK phosphorylation and SIRT1 expression levels. (a and b) AMPK phosphorylation (c) and SIRT1 (d) levels were upregulated after BDMC treatment. *P < 0.05 vs control group; ^#^ *P* < 0.05 vs Aβ_1–42_-induced group from five independent experiments.

Compared with the control group, BDMC treatment did not increase the AMPK phosphorylation levels, and the AMPK phosphorylation levels were decreased in response to Aβ_1–42_ treatment. Compared with the Aβ_1–42_-induced group, BDMC significantly increased the AMPK phosphorylation levels (Figure 1a and b). A similar pattern for SITR1 expression levels was observed (Figure 1c and d).

### 3.2 BDMC enhances the cell survival rate of SK-N-SH cells treated with Aβ_1–42_ via AMPK and SIRT1

To investigate the effects of BDMC on the cell survival of SK-N-SH cells induced by Aβ_1–42_, the cell viability was evaluated after the cells were pretreated with BDMC and with or without compound C or EX527 and co-cultured with Aβ_1–42_ for 24 h.

**Figure 2:**
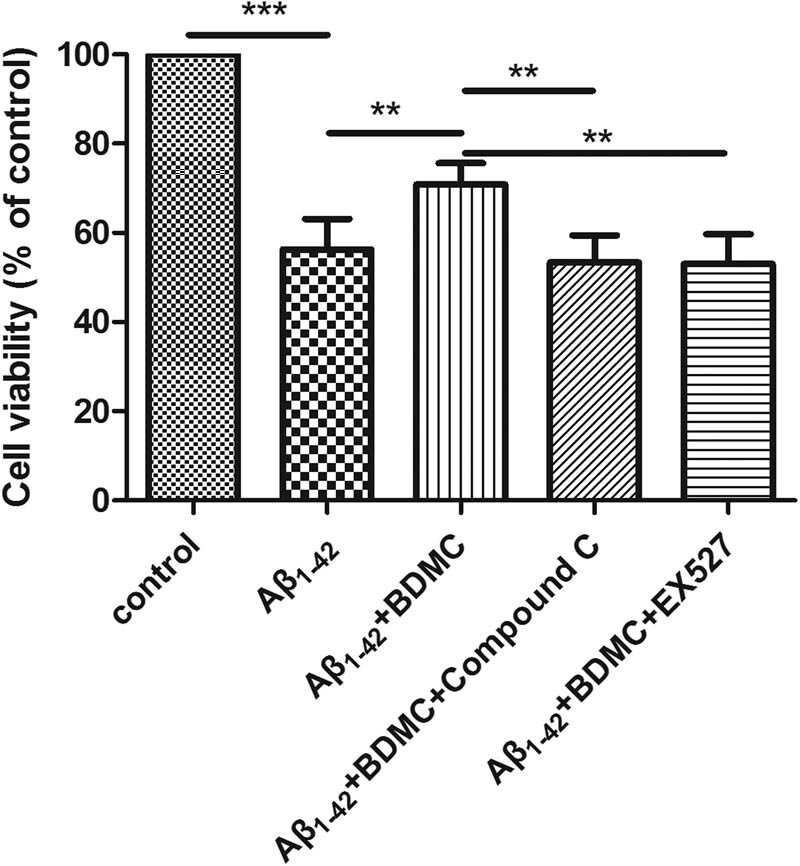
Effects of BDMC on the cell survival in SH-SY5Y cells treated with SK-N-SH cells induced by Aβ_1–42_ after inhibition of the AMPK/SIRT1 signaling pathway. A total of 1 × 10^4^ SH-SY5Y cells were pretreated with 10 µM compound C (an AMPK signaling pathway inhibitor) or 100 nM EX527 and 15 µM BDMC for 30 min before 24 h co-culture with Aβ_1–42_. The cell survival rate is upregulated in SK-N-SH cells induced by Aβ_1–42_ in response to BDMC treatment, but did not increase upon inhibition of the AMPK/SIRT1 signaling pathway. **p < 0.01 and ***P < 0.01 from five independent experiments.

Compared with the control group, the cell survival rate decreased in response to Aβ_1–42_ treatment. Compared with the Aβ_1–42_-induced group, BDMC significantly increased the cell survival rate. However, following AMPK and SIRT1 inhibition, BDMC did not increase the cell survival rate (Figure 2).

### 3.3 BDMC enhances the SOD levels of SK-N-SH cells treated with Aβ_1–4_ via AMPK and SIRT1

To investigate the effects of BDMC on the SOD activity of SK-N-SH cells induced by Aβ_1–42_, SOD activity was evaluated after the cells were pretreated with BDMC and with or without compound C or EX527 and co-cultured with Aβ_1–42_ for 24 h.

**Figure 3:**
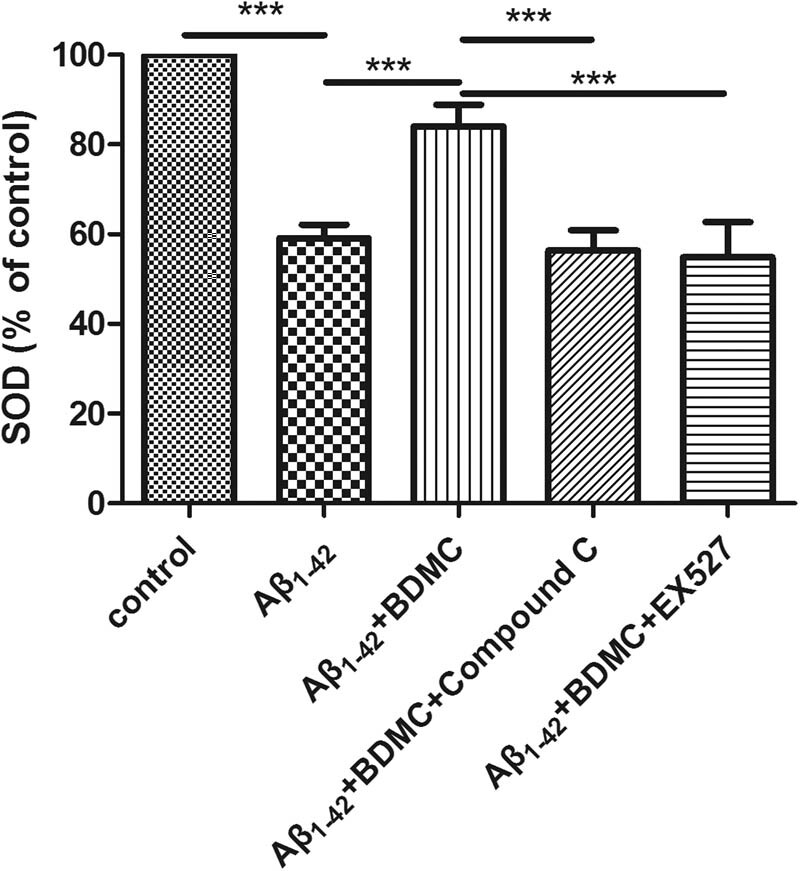
Effects of BDMC on SOD levels in SH-SY5Y cells treated with SK-N-SH cells induced by Aβ_1–42_ after inhibition of the AMPK/SIRT1 signaling pathway. A total of 1 × 10^4^ SH-SY5Y cells were pretreated with 10 µM compound C (an AMPK signaling pathway inhibitor) or 100 nM EX527 and 15 µM BDMC for 30 min before a 24 h co-culture with Aβ_1–42_. SOD levels are upregulated in SK-N-SH cells induced by Aβ_1–42_ in response BDMC treatment, but did not increase upon inhibition of the AMPK/SIRT1 signaling pathway.^***^ *P* < 0.01 from five independent experiments.

Compared with the control group, SOD levels were decreased in response to Aβ_1–42_ treatment. Compared with the Aβ_1–42_-induced group, BDMC significantly increased the SOD levels. However, after AMPK and SIRT1 inhibition, BDMC did not increase the SOD levels (Figure 3).

### 3.4 BDMC enhances the GSH levels of SK-N-SH cells treated with Aβ_1–42_ via AMPK and SIRT1

To investigate the effects of BDMC on the GSH levels of SK-N-SH cells induced by Aβ_1–42_, GSH levels were evaluated after the cells were pretreated with BDMC and with or without compound C or EX527 and co-cultured with Aβ_1–42_ for 24 h.

**Figure 4:**
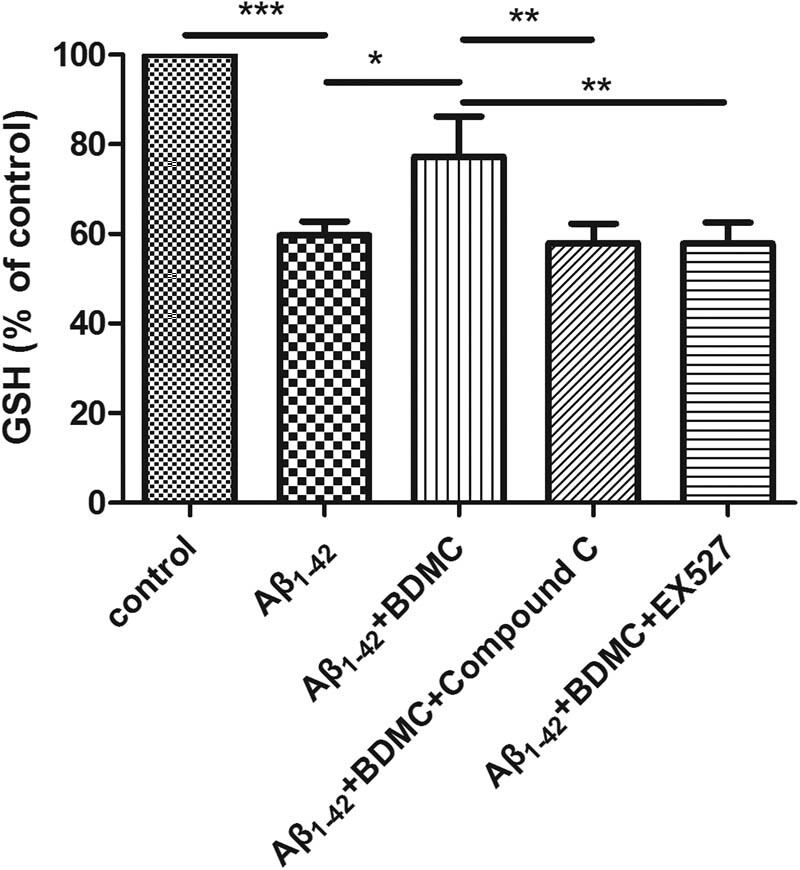
Effects of BDMC on GSH levels in SH-SY5Y cells treated with SK-N-SH cells induced by Aβ_1–42_ after inhibition of the AMPK/SIRT1 signaling pathway. A total of 1 × 10^4^ SH-SY5Y cells were pretreated with 10 µM compound C (an AMPK signaling pathway inhibitor) or 100 nM EX527 and 15 µM BDMC for 30 min before 24 h co-culture with Aβ_1–42_. GSH levels are upregulated in SK-N-SH cells induced by Aβ_1–42_ in response to the treatment of BDMC but did not increase upon inhibition of the AMPK/SIRT1 signaling pathway. ***P < 0.01 from five independent experiments.

Compared with the control group, the GSH levels were decreased in response to Aβ_1–42_ treatment. Compared with the Aβ_1–42_-induced group, BDMC significantly increased the GSH levels. However, after AMPK and SIRT1 inhibition, BDMC did not increase the GSH levels (Figure 4).

## 4 Discussion

In this study, we revealed that BDMC enhanced the cell survival, anti-OS ability and the levels of AMPK phosphorylation and SIRT1 in SK-N-SH cells treated with Aβ_1–42_. However, after inhibition of the AMPK/SIRT1 signaling pathway, BDMC cannot exert these neuroprotective roles. This study demonstrated that BDMC protected SK-N-SH cells from the neurotoxicity induced by the Aβ_1–42_ treatment via the AMPK/SIRT1 signaling pathway.

The accumulation of Aβ peptides was identified as a key step in the multiple pathogenic changes involved in neurodegeneration and dementia [42,43]. Aβ peptide fragments were observed to directly or indirectly induce neuronal cell death [44,45,46]. *In vivo*, small, stable oligomers of Aβ_1–42_ have been isolated from the brain, plasma and cerebrospinal fluid [47,48,49] and correlated with the severity of neurodegeneration in AD [50,51]. Previous studies demonstrated that neurotoxicity induced by Aβ_1–42_ can lead to the apoptotic cell death [52,53]. Therefore, the inhibition of neuronal apoptosis induced by Aβ protein provides a feasible method for the prevention and treatment of AD. In the present study, we observed that BDMC can decrease the cell death of SK-N-SH cells induced by Aβ_1–42_.

One of the most well-known and studied effects of Aβ is its capacity to induce, and be induced by, OS; thus, Aβ induces OS *in vivo* and *in vitro* [54,55,56,57]. OS is able to induce the increased production of Aβ [58,59,60]. A previous study reported that the accumulation of intraneuronal Aβ oligomers resulted in the dysfunction of mitochondria and synapses after neurotoxicity; moreover, mitochondria-targeted antioxidants can protect against the neurotoxicity of Aβ in AD [61,62,63]. SOD and GSH are important antioxidants by scavenging oxygen free radicals against OS. SOD is a metal enzyme that can catalyze the dismutation of superoxide anions, scavenge O_2_^−^ and repair damaged cells. It is an important enzyme that can scavenge free radicals in the brains of AD mice [64]. SOD levels directly reflect the degree of senescence in mice brain cells [65]. The profound role of GSH as a detoxifying agent in the brain is critically important [66]. GSH is also involved in other cellular processes such as neuroinflammation and ferroptosis, which brings the attention of pharmacologists pertaining to medical interventions for therapeutic benefits. Depleted levels of GSH trigger ROS generation implicated in the cell death that causes various neurological diseases like AD [67]. In the present study, we observed that BDMC can increase SOD and GSH levels of SK-N-SH cells induced by Aβ_1–42_.

AMPK can modulate α and β-secretases expression, thus influencing Aβ generation and APP processing [68]. Upon inflammation, the combination of AMPK with SIRT1could exert synergistic effects to jointly maintain energy homeostasis [69,70]. A decline in the SIRT1 activity in mice could be related to oxidative damage [71]. In this study, we observed that the phosphorylation of the AMPK/SIRT1 signaling pathway was upregulated in response to BDMC treatment, whereas BDMC cannot exert any functional roles after inhibiting this pathway.

To conclude, this study found that BDMC alleviated the neurotoxicity of rotenone in SK-N-SH cells by improving cell survival and anti-OS. These effects might be exerted via inhibiting the AMPK/SIRT1 signaling pathway. This may lay the foundation for BDMC to be a novel strategy for the treatment of AD.

Despite the results, this study still has some limitations. Further studies, including TUNEL assays, are required to investigate the modulating roles of BDMC on free radicals and cell death in treated cells under the pathological conditions of AD.
